# Visualization of risk of radiogenic second cancer in the organs and tissues of the human body

**DOI:** 10.1186/s13014-015-0404-x

**Published:** 2015-04-28

**Authors:** Rui Zhang, Dragan Mirkovic, Wayne D Newhauser

**Affiliations:** Mary Bird Perkins Cancer Center, LA, Baton Rouge USA; Medical Physics Program, Department of Physics and Astronomy, Louisiana State University, LA, Baton Rouge USA; Department of Radiation Physics, The University of Texas MD Anderson Cancer Center, Houston, TX USA

**Keywords:** Radiogenic second cancer, Risk, Visualization, Volumetric modulated arc therapy, Proton therapy

## Abstract

**Background:**

Radiogenic second cancer is a common late effect in long term cancer survivors. Currently there are few methods or tools available to visually evaluate the spatial distribution of risks of radiogenic late effects in the human body. We developed a risk visualization method and demonstrated it for radiogenic second cancers in tissues and organs of one patient treated with photon volumetric modulated arc therapy and one patient treated with proton craniospinal irradiation.

**Methods:**

Treatment plans were generated using radiotherapy treatment planning systems (TPS) and dose information was obtained from TPS. Linear non-threshold risk coefficients for organs at risk of second cancer incidence were taken from the Biological Effects of Ionization Radiation VII report. Alternative risk models including linear exponential model and linear plateau model were also examined. The predicted absolute lifetime risk distributions were visualized together with images of the patient anatomy.

**Results:**

The risk distributions of second cancer for the two patients were visually presented. The risk distributions varied with tissue, dose, dose-risk model used, and the risk distribution could be similar to or very different from the dose distribution.

**Conclusions:**

Our method provides a convenient way to directly visualize and evaluate the risks of radiogenic second cancer in organs and tissues of the human body. In the future, visual assessment of risk distribution could be an influential determinant for treatment plan scoring.

## Background

Radiation therapy has long been used as an effective treatment for malignancies in cancer patients. However, for many patients, the late effects of radiation (e.g., second cancers, cardiac toxicities, reductions in fertility, bone growth abnormalities, and cognitive deficits) reduce their survival time and/or quality of life after treatment [1-3]. With increasing long-term survival rates in cancer patients [4], avoiding treatment-related late effects is increasingly important [5]. Second cancers are of particular concern because of their high incidence and they account for about 16% of all cancers in the United States [6]. A majority of second cancers are malignant and these are difficult to control [7]. To develop strategies to minimize radiogenic second cancer, we must first be able to routinely predict patients’ risks of developing second cancers, which has not become feasible until recently because of limitations in dose reconstruction methods and dose-risk models [8].

Currently, clinicians evaluate the quality of a radiation therapy by inspection of the treatment plan generated by modern treatment planning systems (TPSs), including prescription dose to the target, dose distribution overlaid on the patient’s images, dose volume histograms, and other dosimetric figures-of-merit, such as the dose homogeneity index [9], conformity index [10], normal tissue complication probability (NTCP) [11], and tumor control probability [12]. Among those measures, only spatial dose distributions can be visually checked in two or more dimensions simultaneously, which provides very valuable information, such as the anatomic location of dose hot and cold spots. The NTCP and other commonly used scalar risk quantities lack spatial information because they only provide a single numerical value. There has been progress in studies of risks of radiogenic late effects in recent years [13-19], but the investigation of the spatial distribution of risks in the human body is limited to a few groups [5,20,21].

In this work, we developed methods to visualize the risk of radiogenic second cancer for patients, taking into account treatment factors (e.g., dose, beam direction) and host factors (e.g., sex and age at exposure). We demonstrated the feasibility of this method by creating treatment plans for two patients undergoing photon and proton therapies using commercial TPSs, converting radiation doses from the TPSs to the risks of radiogenic second cancer using dose-risk models from the literature, and displaying the risk distributions superimposed on the patient’s computed tomography (CT) images. The limitations and uncertainties associated with risk visualization were also examined.

## Methods

### Patients and treatment techniques

The first patient was a 67-year-old man diagnosed with moderately differentiated adenocarcinoma of the prostate, received prostatectomy and treated with volumetric modulated arc therapy (VMAT) at Mary Bird Perkins Cancer Center. Three-dimensional CT images were acquired with 2.5-mm thick slices from the waist to the thigh. The VMAT plan was created using a commercial TPS (Pinnacle, Philips Medical Systems, Fitchburg, WI). The dose prescription was 68 Gy administered in 2 Gy/fraction to the prostate bed. Two 6-MV overlapping 350° arcs were utilized, with a 45° collimator angle for both. The organs at risk for this patient included the bladder, rectum, prostate, and remainder (i.e., all other tissues/organs) as specified by the report of the committee on the Biological Effects of Ionizing Radiation (BEIR VII) [22]. Because the risk coefficient was provided for the whole colon in BEIR VII and the rectum is only part of the colon and because only part of the whole body was scanned, the risks for the rectum and remainder were scaled down by mass fractions as we previously reported [23], i.e., we divided the mass of rectum by the mass of whole colon, and divided the mass of remainder in the current CT data by the mass of total body which can be obtained from patient’s record.

The second patient was a 13-year-old girl diagnosed with medulloblastoma and treated with surgical resection and passively scattered proton therapy at the University of Texas MD Anderson Cancer Center. The patient was treated with craniospinal irradiation (CSI) of 21.3 Gy administered in 1.64 Gy/fraction. A CT scan with 2.5-mm-thick slices was obtained from the top of the head to the thigh. The proton treatment plan was created using a commercial TPS (Eclipse, Varian Medical Systems, Palo Alto, CA). The plan contained five fields: right and left posterior oblique cranial fields and three posterior-anterior spinal fields. The organs of interest included stomach, colon, lungs, bladder, thyroid, breast, liver, uterus, ovary, and remainder. The risk for the remainder was scaled down by a mass fraction because the CT scan did not include anatomy below the thigh.

Both patients’ data were exported from TPS and anonymized [24] for research purpose. Only primary doses reported by TPS were used in risk estimations.

### Risk estimation and visualization

In the traditional approach cancer risk was estimated from radiation exposure for radiation protection purpose, which has been adopted by several authors for second cancer risk estimation after radiation therapy [25,26], the risk of second cancer for each organ/tissue was calculated as

1 $$ {R}_T={\overline{H}}_T\times {\left(\frac{R}{H}\right)}_T={\overline{w}}_R{\overline{D}}_T\times {\left(\frac{R}{H}\right)}_T, $$

where *R*_T_ is the risk in tissue T, ${\overline{H}}_T$ is the mean equivalent dose in tissue T, ${\overline{D}}_T$ is the mean organ dose calculated by the TPS and $\overline{w_R}$ is the mean radiation weighting factor (1.1 for proton beams and 1 for photon beams). The reasons to use 1.1 for proton radiation weighting factor are: 2 was recommended for general use and included very high proton energies near 1 GeV from cosmic radiation [27,28]. While for proton therapy, the highest energy is around 200 MeV and the LET for these protons is less than 10 keV/μm [27]. The mean quality factor is around 1.1 for 150 MeV protons [27,28]. Taking these into account, we think it is more appropriate to use 1.1 than 2 as the mean radiation weighting factor for therapeutic protons. ${\left(\frac{R}{H}\right)}_T$ is the organ-specific relative or absolute risk coefficient taken from tables 12D-1 in the published BEIR VII report [22] and the dose and dose-rate reduction (DDREF ) factor (1.5) was taken out because we are studying risks after high dose radiotherapies. For brevity, we discuss only lifetime absolute risk of cancer incidence in this paper although the methods could be applicable to arbitrary time point and endpoints.

**Figure 1:**
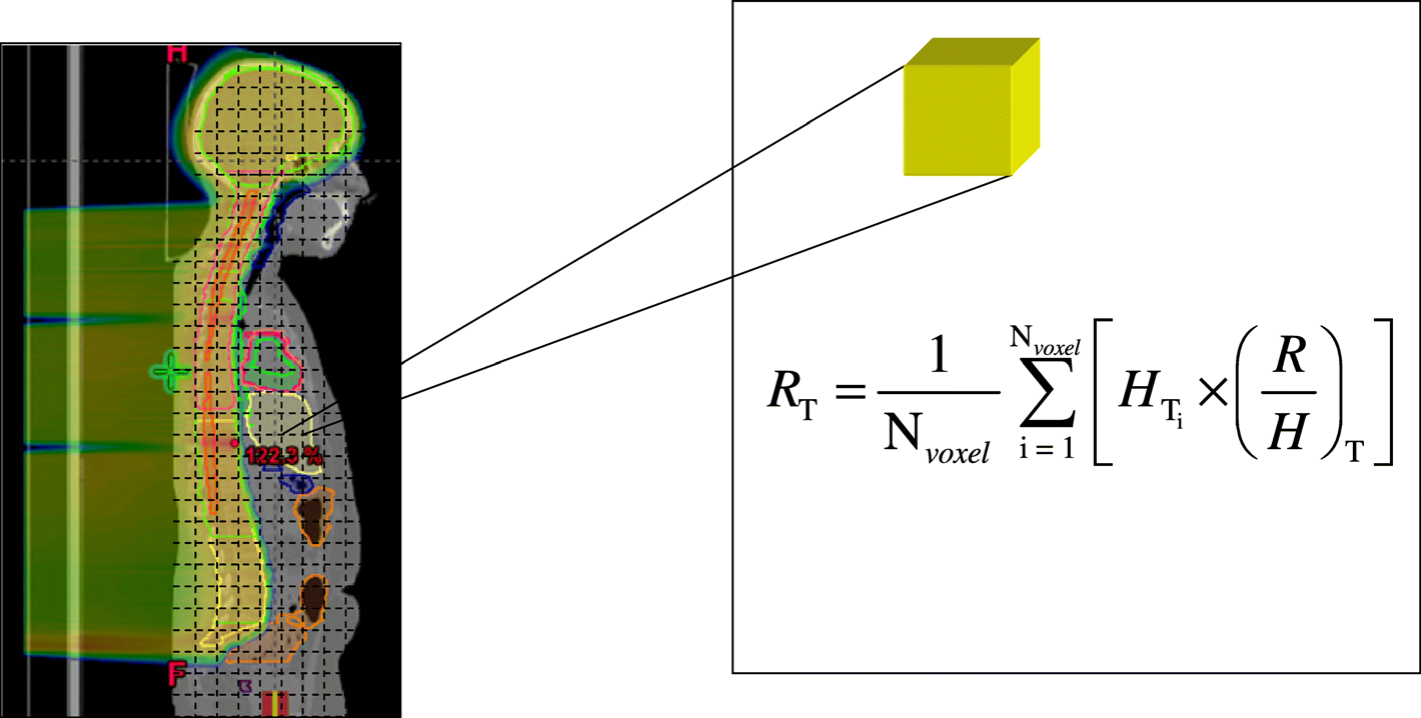
Schematic illustration of risk estimation method (using LNT dose-risk model) based on a voxelized phantom.

However, Dorr and Herrmann [29] and Diallo *et al* [30] reported most of the second cancer tumors were localized within the border of the irradiated volume instead of uniformly distributed within the organ/tissue. These findings showed that a mean organ dose should not be used to calculate risk of second cancer when there is a dose heterogeneity within the organ. We calculated risk using an in-house code (based on MATLAB software, Mathworks, version 7.9, Natick, MA) and a voxelized phantom based on each patient’s data (Figure 1).

**Figure 2:**
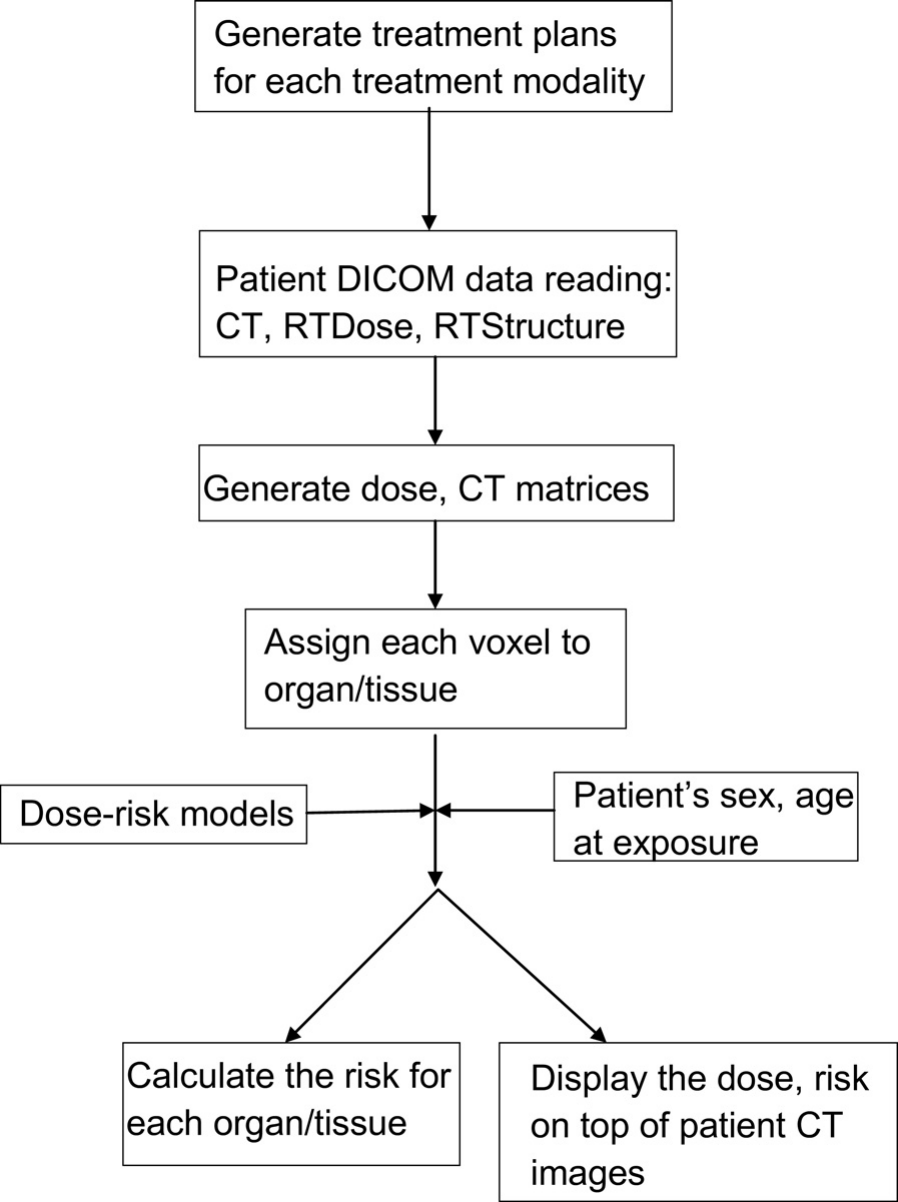
Flow chart for risk estimation and visualization.

To accomplish this, we performed the following steps: First, the patient-related data, including CT images, RTDose and RTStructure files were read in Digital Imaging and Communication in Medicine (DICOM) format by the in-house code. The code generated three-dimensional dose and CT matrices. Second, each voxel in the three-dimensional dose matrix was uniquely assigned to one organ/tissue by comparing the coordinates of that voxel with the coordinates of organs in the RTStructure file which contains all the structure contour information. Third, by applying a linear non-threshold (LNT) dose-risk model, taking into account patient’s sex and age at exposure, the risk to the whole organ, *R*_T_, was calculated according to

2 $$ {R}_{\mathrm{T}}={\displaystyle \sum_{i=1}^{N_{\mathrm{T}, voxel}}\frac{H_{\mathrm{T}i}}{N_{\mathrm{T}, voxel}}\times {\left(\frac{R}{H}\right)}_{\mathrm{T}}}, $$

where *H*_Ti_ is the equivalent dose in the *i*th voxel, and *N*_T,*voxel*_ is the total number of voxels in the organ. The spatial distribution of the risk was superimposed on the CT images as a color overlay. Figure 2 schematically shows the methods for risk estimation and visualization.

Dose-risk relationships are uncertain below approximately 50 mSv and above approximately 2.5 Sv, e.g., at high therapeutic doses. At low doses, we shall assume a LNT behavior. At high doses, cell sterilization mechanisms may be effective, and some dose-risk relationships (e.g., linear-plateau [LPLA] and linear-exponential [LEXP] relationships [21,31,32]), may describe the radiobiological outcomes better than a linear model. For example, there is strong evidence that the dose-risk relationship for thyroid is not linear [33-35]. Therefore, in order to explore the possible influence of non-linear behaviors on risk visualization, these alternative dose-risk models were also applied in addition to the LNT model used in the BEIR VII [22] when we calculated and displayed risks. The LNT model was used as a baseline model. For the LPLA model, the risk was calculated as

3 $$ {R}_{\mathrm{T}}={\displaystyle \sum_{i=1}^{N_{\mathrm{T}, voxel}}\frac{1}{N_{\mathrm{T}, voxel}\delta }{\left(\frac{R}{H}\right)}_T\left(1-{e}^{-\delta {D}_{\mathrm{T}i}\times RB{E}_m}\right),} $$

where δ is a cell sterilization parameter, *D*_Ti_ are the physical dose in the *i*th voxel and RBE_m_ is the RBE defined for cell killing deterministic effects [27] and is 1 for photon and 1.1 for proton [36]. For the LEXP model, the risk was calculated as

4 $$ {R}_{\mathrm{T}}={\displaystyle \sum_{i=1}^{N_{\mathrm{T}, voxel}}\frac{1}{N_{\mathrm{T}, voxel}}{\left(\frac{R}{H}\right)}_T{H}_{\mathrm{T}i}{e}^{-\alpha {D}_{\mathrm{T}i}\times RB{E}_m},} $$

where α is a cell sterilization parameter. Numerical values for α and δ were determined heuristically to obtain the correct form, i.e., they were iteratively adjusted so the dose response curves will bend at certain dose value [14,37].

Unlike absorbed dose (an intrinsic quantity), the spatial visualization of risk (an extrinsic quantity) distributions depends on the voxel size used. The “absolute risk” in any voxel could be very small if there are a large number of voxels in the organ that voxel belongs to because the risk coefficient is defined for the whole organ, and ${\left(\frac{R}{H}\right)}_{\mathrm{T}}/{N}_{T, voxel}$ would be small if *N*_T,*voxel*_ is large. Although this does not affect the final whole-organ risk, it can complicate the visualization of risk distributions. This “voxel-size problem” increases in severity with increasing organ size, decreasing ${\left(\frac{R}{H}\right)}_{\mathrm{T}}$, decreasing voxel size and decreasing dose. To overcome this problem, we color-coded risks by organ and visualized spatial distributions of “risk gradient” by the degree of transparency of the risk colorwash.

## Results

**Figure 3:**
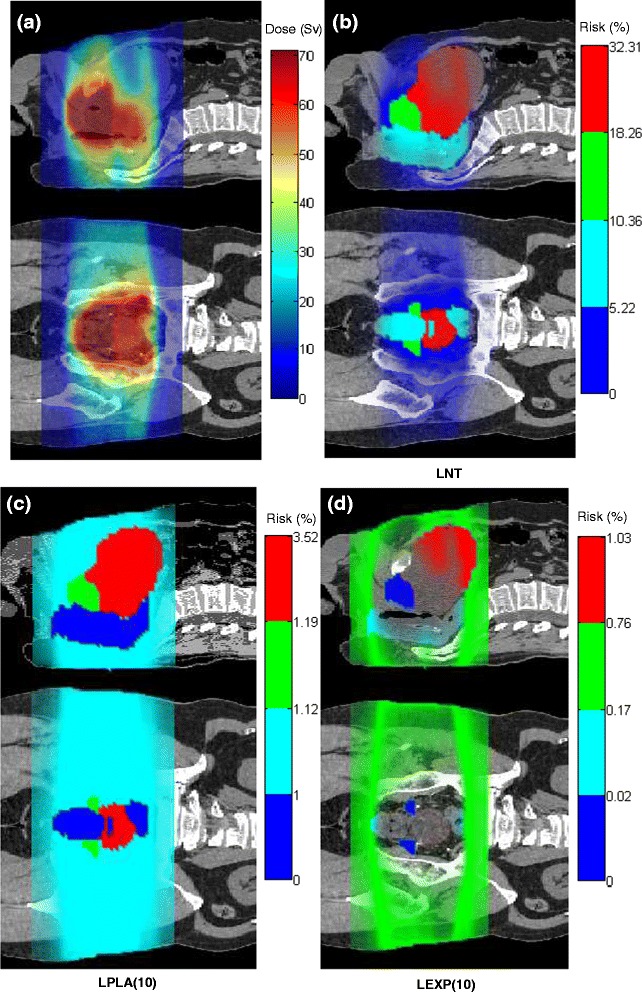
Visualization of **(a)** equivalent dose and **(b)**, **(c)**, and **(d)** lifetime risks of second cancer incidence based on different dose-risk relationships (LNT: linear non-threshold model, LPLA(10): linear plateau model with bending point at 10 Sv, LEXP(10): linear exponential model with bending point at 10 Sv) on sagittal and coronal slices for a 67-year-old man who received photon VMAT prostate treatment.

Figure 3 shows the distributions of equivalent doses and lifetime risks of incidence of second cancer based on different dose-risk relationships (LNT, LPLA(10), and LEXP(10)); the number in the parentheses refers to 10 Sv, the value beyond which risk becomes non-linear with dose, following the methods of previous study [37]. When we used the LNT model, the predicted lifetime risks of second cancer incidence were 32.3% for the bladder, 18.3% for the prostate, 10.4% for the rectum, and 5.2% for the remainder (Figure 3b). The risk distributions were starkly different from the dose distributions (Figure 3a) for all the risk models considered. Risks were high in high-dose regions when the LNT model was applied, whereas risks decreased in the high-dose regions when the LPLA or LEXP models were applied, and this was especially true for the LEXP(10) model (Figure 3d). For example, in the bladder, the risk distributions were reversed when the LEXP(10) model replaced the LNT model because risk decreased after 10 Sv, whereas the risk distribution in the bladder was almost uniform when LPLA(10) was used because the risk plateaued after 10 Sv (Figure 3c).

**Figure 4:**
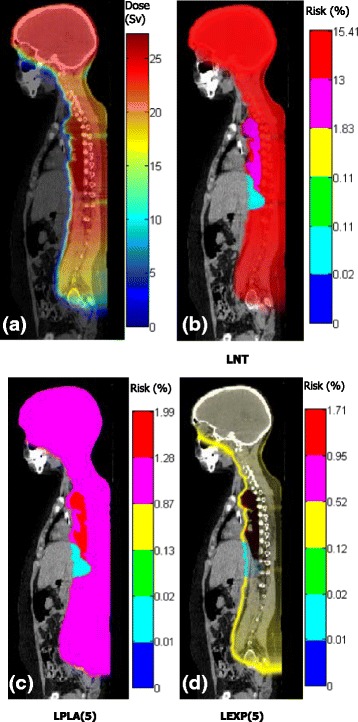
Visualization of **(a)** equivalent dose and **(b)**, **(c)**, and **(d)** lifetime risks of second cancer incidence based on different dose-risk relationships (LNT: linear non-threshold model, LPLA(5): linear plateau model with bending point at 5 Sv, LEXP(5): linear exponential model with bending point at 5 Sv) on a sagittal slice for a 13-year-old girl who received proton CSI.

Figure 4 shows the distributions of the doses and lifetime risks of incidence of second cancer based on dose-risk relationships (LNT, LPLA [5], LEXP [5]); the number in the brackets is the equivalent dose in unit of Sv, the value beyond which risk becomes non-linear with dose, which was chosen for the second patient who received proton CSI (some organs of interest cannot be seen on the slices presented here) follow the methods in previous study [14]. According to the LNT model, the lifetime risks were 15.4% for the remainder, 13% for the lung, 1.8% for the thyroid, 0.1% for the colon, 0.1% for the liver, and 0.02% for the stomach (Figure 4b). The risks for bladder, breast, uterus and ovary were 0 because doses to those organs were 0. When the LNT or LPLA (Figure 4c) model was applied, the shapes of the dose and risk distributions were similar, where the differences were caused by variations in the radiosensitivity of various organs of tissues. However, the risk distribution was very different from dose distribution when the LEXP model was applied when risks decreased after 5 Sv (Figure 4d).

## Discussion

In this study, a method was developed to visualize the predicted risk of radiogenic second cancer for patients who received radiation therapy: one patient received photon VMAT and the other received proton CSI. The dose distributions were extracted from treatment plans, and risks were calculated and superimposed on the patients’ CT images.

The apportionment of whole-organ risk to voxelized subvolumes of the organ according to Eq. (2), (3) and (4) is the simplest and most consistent approach to generalize the whole-organ risk models used from the epidemiology literature. This very simple apportionment approach does not attempt to describe possible variations in myriad and complex mechanisms in carcinogenesis that may occur across an organ or tissue. Stated another way, we did not attempt to model spatial variations in risk coefficients themselves across an organ or tissue. Strictly speaking, before new risk models with finer spatial resolution come out, one cannot calculate risk in each voxel. Rather, for the intents and purposes of this work, we used widely used risk models and simple risk apportionment methods to learn about how radiation dose gradients influence the visualization of risk. In particular, we focused on a vexing problem related to visualizing spatial distributions of risk gradient, which are extrinsic. That said, it is possible that radiation sensitivity varies across an organ or tissue, e.g., due to variations in concentration of potential clonogens, oxygen, repair capacities, and other factors. In principle, if such dependencies can be observed and modeled, this could be used to refine the risk apportionment method in this study. To our knowledge, there is insufficient knowledge to implement such refinements at this time.

Some earlier studies, all emanating from the same group [20,21,38], utilized a similar method to show second cancer risk distribution in the regions where second cancers had developed. Basically they showed a normalized relative risk map in different regions (organs) while we showed absolute risks. As they pointed out in their paper [20] and as we mentioned in our manuscript, their way of displaying risk distribution suffered from the “voxel-size problem” because the “voxelized risk” value for a larger organ will be small and difficult to visualize. Again, due to the limitation of current dose-risk models, the definition of “voxelized risk” has a conceptual problem. To overcome this limitation, in the present study, the risks were color-coded by organ and spatial distribution of risk gradient was visualized by the degree of transparency of the risk colorwash. In this way, the organ specific risks can be assessed quickly (each organ has a different color) and detailed assessment of sub-regions of the individual organs can be visualized clearly. Regardless of the differences between our study and theirs, their qualitative finding was consistent with ours in that the risk was concentrated at the edges of the high-dose region when the LEXP model was used, and the risk was concentrated in the high-dose area when the LPLA model was used.

Dorr and Herrmann [29] studied a sample of patients who developed second tumors from a cohort of about 31000 cancer patients who received radiotherapy in Germany between 1969 and 1989. They reported that most of the second tumors occurred in volumes receiving < 6 Gy and were seen within the margin region of the treatment volume (2.5 cm inside to 5 cm outside the 50% isodose line). Diallo et al [30] also studied a sample of patients with second cancers from a cohort of 4581 patients who were irradiated between 1942 and 1986 at 8 French and United Kingdom centers, and they reported that the majority of second solid cancers appeared in low- or intermediate-dose regions (<2.5 Gy) bordering the irradiated volume. Based on this information and the risk distributions for the 2 patients in this study, it appears possible that the LEXP model may provide the qualitatively closest result compared with aforementioned observational evidence. On the other hand, epidemiologic study like Berrington de Gonzalez et al [39] reported that second cancer risk kept increasing even at organ doses higher than 60 Gy, except thyroid cancer which clearly showed a downturn after 20 Gy. Further studies still need to be carried out to test these findings. Our risk visualization methods and software platform can be used for research on this and other aspects of risk prediction and visualization.

One limitation of this work was the availability of dose-risk models that are valid at therapeutic doses. Currently, there is no consensus on what model should be used for organs or tissues that receive high primary radiation doses except thyroid. Many researchers have argued that the BEIR VII models and other similar models were developed for low doses and low dose rates and some have proposed alternative dose-risk models [40-43]. However, these new risk models are either still under development or less well established and validated. The “voxel-size problem,” which we mentioned in the methods section of this report, is also due in part to the limitation of available risk models which only provide risk coefficients for whole organs. And as Pfaffenberger et al [20] pointed out, it is problematic to scale down the whole organ risk to risk in one voxel using individual size of the patient’s organ while the risk coefficients was defined using a mean organ size. Risk models with finer spatial resolution and improved accuracy are needed. Development of the latter will require large samples of radiation therapy patient data for which accurate dose distributions and cancer incidence are available. Furthermore, our results suggest that the extrinsic nature of risk distributions and the uncertainties in risk models comprise significant research challenges that must be addressed before risk visualization methods are adopted as clinical decision support tools. However, the basic risk visualization methodology proposed in this study is applicable to arbitrary risk models, and addition risk models can be incorporated into our code system in a straightforward manner. The other limitation in this study is that we assumed tissue density in each voxel is the same within an organ. This is not a serious limitation for the purposes of this study because density variation within one organ is small for most organs except lung, and an enhancement of the code by including three-dimensional voxel density information is in progress. In the future, when risk models with finer spatial resolution are developed or cell level risk coefficients are available, not only tissue density but also tissue/cell type need to be taken into account for risk estimations.

The strengths of this study include the fact that we investigated clinically-relevant advanced-technology radiation treatment modalities and techniques. We used realistic patient data from the two most commonly used commercial TPSs in different institutions. The results of this study are significant because they conceptually demonstrate that risks of radiogenic side effects cannot be assessed by visualizing dose distributions and offer direct spatial information about risks, which cannot be obtained from single mean organ risk values or other scalar quantities. Eventually, the visualization of risk distributions in the human body may become an essential part of the treatment planning, particularly for young patients with good prognoses for long term survival.

Despite the importance of second cancer, the short-term treatment outcome - the primary cancer control - should not be compromised by the consideration of late effects. The primary cancer control remains the highest priority. However, the risks of late effects are often neglected in contemporary treatment planning methods. Risk evaluation of late effects is especially important for pediatric patients who have good prospect to survive their primary cancer but may suffer from radiogenic late effects. Calculating and displaying the risks of other late effects, such as cardiac toxicity etc., would be the next logical step. In addition, besides primary dose, stray radiation doses should be included because most second cancers occur in low- or medium-dose areas [29,30]. Monte Carlo techniques [13], measurements [44], or analytical models [45,46] should be used to assess stray doses because current TPSs cannot calculate them accurately. Although color scales and degrees of transparency were used in this paper, “iso- risk gradient” lines could also be used in a similar manner, i.e., using solid line for the highest risk, dashed line for the second highest risk, dot dashed for the third highest risk, and so on. Our method for risk estimation and visualization can be incorporated into current commercial TPSs in a straightforward manner and could in principle be used for personalized patient risk assessments, algorithmic optimization of treatment plans by taking into account radiogenic late effects [47], and to supplement the physicians’ judgment of treatment modality selection.

## Conclusion

In summary, we have developed and demonstrated a new method to visualize the risk of radiogenic second cancers. This tool allows direct visualization and quantitative assessment of the risks of radiogenic second cancer, including the mean organ risk, spatial distribution of risk within organs, and possible variations of the risk value and distribution by taking uncertainties into account. It also revealed the current limitations of the risk estimation due to the extrinsic nature of the spatial risk distributions and non-linearity in dose response models. It may also be used to guide the development of future dose-risk models, and predict the distribution of second cancer and other radiogenic late effects after both conventional and modern radiation treatments.

### Consent

Written informed consent was obtained from the patient for the publication of this report and any accompanying images.
